# Evaluating the Functional Outcomes and the Quality of Life for Pediatric Patients with Osteogenesis Imperfecta after Fracture Treatment with Intramedullary Rodding

**DOI:** 10.3390/children8111066

**Published:** 2021-11-19

**Authors:** Alexandru Herdea, Alexandru Ulici, Dimitra Qirjako, Alexandra Toma, Răzvan Petru Derihaci, Claudiu N. Lungu, Adham Charkaoui

**Affiliations:** 1 11th Department, “Carol Davila” University of Medicine and Pharmacy, 050474 Bucharest, Romania; alexherdea@yahoo.com (A.H.); qirjakodimitra@gmail.com (D.Q.); 2 Pediatric Orthopedics Department, “Grigore Alexandrescu” Children’s Emergency Hospital, 011743 Bucharest, Romania; 3 Department of Morphological and Functional Sciences, Faculty of Medicine and Pharmacy, “Dunărea de Jos” University of Galați, 800008 Galați, Romania; dr.alexandratoma@gmail.com (A.T.); charkaoui.adham@gmail.com (A.C.); 4 Department of Gynecology and Obstetrics, University Carl Gustav Carus, 01307 Dresden, Germany; razvanpetru.derihaci@uniklinikum-dresden.de; 5 Department of Surgery, Country Emergency Hospital Brăila, 810249 Brăila, Romania; lunguclaudiu5555@gmail.com

**Keywords:** osteogenesis imperfecta, functional outcomes, quality of life, telescopic rod, pediatric orthopedics

## Abstract

Background and Objectives: Osteogenesis imperfecta is a rare pathology involving the bones and the connective tissues, generating alterations that lead to frequent fractures during childhood. When fractures occur at birth, they are associated with an impairment of walking and the quality of life. Although surgical techniques have significantly improved in recent years, functional outcomes and the quality of life for pediatric patients that benefited from surgical management with telescopic rods have been less evaluated. This study aimed to measure functional results and determine the factors that influence the quality of life for the pediatric population diagnosed with Osteogenesis imperfecta and surgically treated using the telescopic rod approach after suffering a fracture or severe deformity. Materials and Methods: We conducted a cohort study that consisted of 15 patients diagnosed with Osteogenesis imperfecta (average age of 11.6 years). All individuals possessed at least one intramedullary telescopic rod as a result of the surgical treatment. Results: We observed that the pain, both acute and chronic, impairs the quality of life and interferes with daily living activities, for instance, self-care tasks. Conclusions: Osteogenesis imperfecta is associated with a severely damaged level of walking. All of the aspects of the pediatric patient’s daily life activity seem to be affected. Furthermore, these patients, especially those residing in rural areas, have a poor quality of life.

## 1. Introduction

Osteogenesis imperfecta (OI) represents a genetically transmitted group of disorders characterized by increased bone fragility and connective tissue dysfunction. The leading cause is the mutation of one of two genes COL1A1 and COL1A2, which are responsible for encoding type I procollagen [1].

Sillence et al. developed the first classification [2]. In 1979, he described four types of Osteogenesis imperfecta based on genetic inheritance, clinical signs and symptoms and also radiological findings:

- mild, nondeforming
- perinatally, lethal
- severe, progressively deforming
- common, moderately severe.

The severity varies from mild to deadly forms, particularly during the perinatal period. This classification has been expanded based on recent advances in Osteogenesis imperfecta’s molecular basis and currently contains 13 types [2].

The alteration of bone biomechanics leads to Osteogenesis imperfecta’s major clinical characteristics, such as repeated fractures occurring mainly at the inferior limbs, osteopenia, and progressive skeletal deformities. Additional clinical manifestations such as blue sclerae, dentinogenesis imperfecta, adult-onset deafness, joint laxity and even hypotonia may also be encountered in the pediatric patients diagnosed with Osteogenesis imperfecta [3]. 

A positive diagnosis is made based on clinical and radiological findings of each pediatric patient. Measuring bone mineral density, other bone metabolic markers, or a DNA test can also help in controversial cases [4,5].

The type of Osteogenesis imperfecta, number of fractures, skeletal deformation, and age of onset define children’s ability to walk and their level of ambulation [6]. Osteogenesis imperfecta type I, the most common type, is associated with mild impairment of motor function. The patients can achieve full motor development and be independent in performing activities of daily living. Almost all patients that belong to Osteogenesis imperfecta type III and IV are limited to an electric wheelchair function later in life, despite the complex treatment and the intensive rehabilitation. Disproportion in stature can be variable and is caused by different factors such as platyspondyly in type I Osteogenesis imperfecta, or significant scoliosis and kyphosis in type III and IV Osteogenesis imperfecta, leading to a reduced final adult height [7].

Early surgical treatment indicated for repeated fractures and deformities, combined with medical treatment represented mainly by bisphosphonates, can enhance motor development and function [1]. 

The impairment of motor function is associated with a deficiency of social cognition and communication. The above-stated are also defining aspects of these patients’ quality of life.

In this study, we are evaluating the functional outcomes in patients with Osteogenesis imperfecta that have been surgically treated after suffering a fracture or having a severe deformity that would lead to a fracture in a matter of time. Furthermore, we determined the predominantly affected areas of the quality of life, comparing the postoperative follow-up results to the preoperative period.

## 2. Materials and Methods

We conducted a study in the Pediatric Orthopedics Department at “Grigore Alexandrescu” Children’s Emergency Hospital in Bucharest, approved by The Ethics Committee from “Grigore Alexandrescu” Children’s Emergency Hospital, Bucharest, Romania. The study is a cross-sectional one, evaluating patients with Osteogenesis imperfecta that have been surgically treated using the telescopic rod approach. The primary endpoint was to evaluate the quality of life for pediatric patients diagnosed with Osteogenesis imperfecta after performing surgical repair of a bone fracture. Evaluation occurred before and after the surgical intervention. The first and foremost aspect, by which the present study debuted, was obtaining the signed consent that was provided from the parents of the study participants and all of the data were anonymized at the source. 

**Figure 1:**
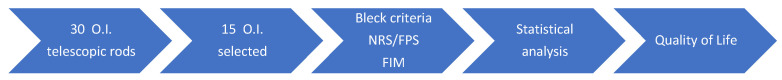
Flow-chart of the study methodology.

**Figure 2:**
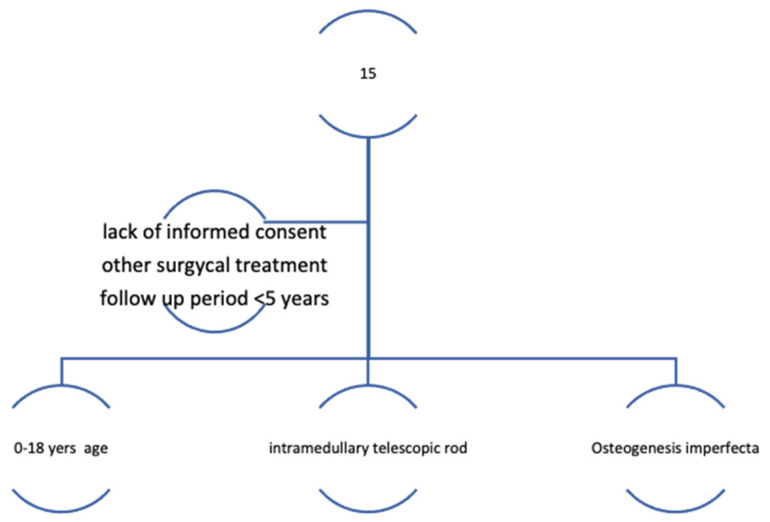
Flow chart of inclusion and exclusion criteria.

Then, using the CONSORT guidelines, a cohort study of 15 patients (eight female patients and seven male patients), whose families agreed to complete the questionnaires, were selected out of a total of 30 patients that underwent surgical treatment by telescopic rods. The average age for the group was 11.6 years (ranging from 4 to 16 years). Following the CONSORT guidelines, patients were first assessed for eligibility (Figure 1 and Figure 2).

Thirty pediatric patients with Osteogenesis imperfecta were considered in the first stage of the study. By applying the inclusion and exclusion criteria, 15 pediatric patients with Osteogenesis imperfecta, that received telescopic rods by surgical approach, were finally selected and followed up for a period of 5 years. In the next stage, a modified Bleck criteria, the Numerical Rating Scale (NRS), the Faces Pain Scale (FPS), and the Functional Independence Measure (FIM) were applied for the previously selected pediatric patients in order to quantify the quality of life for each individual. In the final phase of the present study, statistical analysis of the questionnaires generated the data that were analyzed in order to get a better perspective regarding the quality of life. 

The inclusion criteria incorporated the following aspects:

− age group 0–18 years for Osteogenesis imperfecta patients
− pediatric patients with Osteogenesis imperfecta that underwent a surgical realignment involving the intramedullary telescopic rod in the lower limbs after suffering a fracture or a severe deformity
− the presence of the informed consent from the parents of the study participants
− follow-up period for a period of at least five years
− sufficient provided data.

The interventions were performed in the same hospital, followed by periodic medical treatment using bisphosphonates.

− The exclusion criteria included the following aspects:
− lack of informed consent
− surgical approach using other methods other than intramedullary telescopic rodding
− follow-up for less than five years, or
− insufficient data provided.

Fifteen patients were excluded from the study. Excluded individuals did not meet the inclusion criteria, had declined to participate, or their medical data were incomplete. These measures were adopted in order to avoid sampling bias, bias in the assignment, omitted variables, and self-serving bias. 

Sampling bias occurs when a population member is more likely to be selected in a sample than others. This type of statistical error limits the generalization power of the study, being a threat to external data validity. However, findings from biased samples can be generalized to populations that share characteristics with the same sample. The target population was clearly defined by the inclusion and exclusion criteria stated before in an attempt to avoid this type of error. 

Assignment bias is observed when experimental groups present different characteristics due to a vicious assignment process. Although this type of error is associated with non-random sampling, it can also intervene in random sampling. To minimize this type of error, a purposive sample technique was used. 

An omitted variable leads to bias and inconsistency of coefficient correlation (that are estimated). To overcome this type of error, questionnaires were regularly used to collect as much data as possible. All variables that satisfy the inclusion criteria were collected. 

Self-serving bias refers to associating internal, personal factors to positive outcomes and external situational factors to adverse outcomes. This type of bias is the hardest to avoid when dealing with self-evaluation questionnaires based on collected data. The Numerical Rating Scale, the Faces Pain Scale, and the Functional Independence Measure questionnaires are well organized and require precise answers to avoid this type of error. 

To assess the patient’s quality of life and to overcome the type of errors discussed previously, such as rare conditions with a limited sample size, we used, for the before and after surgery aspects, three different questionnaire-based instruments:

− modified criteria of Bleck [8] to classify the level of ambulation
− the “Numerical Rating Scale” (NRS), or the “Faces Pain Scale” (FPS) to determine the level of pain [9].
− and a disability questionnaire—the FIM (Functional Independence Measure) [10].

The information obtained was reported to the patients’ demographic data. 

The modified Bleck criteria classifies the level of ambulation on a 9-point scale like the following:

1. Non-walker older than two years of age;
2. Therapy walker with the use of crutches or canes;
3. Therapy walker without the use of crutches or canes;
4. Household walker with the help of crutches or canes;
5. Household walker without the use of crutches or canes;
6. Neighborhood walker with the use of crutches or canes;
7. Neighborhood walker without the use of crutches or canes;
8. Community walker with the help of crutches or canes;
9. Community walker without the use of crutches or canes [11].

Community walking achievement is essential, especially for type I of OI, and household or therapy walking for type III and IV [12].

As a common occurrence for children with Osteogenesis imperfecta, pain is also an important aspect worth evaluating regarding the quality of life. Pain can be acute, related to a new fracture, or chronic, such as the one identified in the old fractures, unstable joints, and muscle pain. 

For pain intensity assessment, we used the Numerical Rating Scale for patients older than ten years, and the Wong-Baker Faces Pain Rating Scale/Faces Pain Scale for pediatric patients between the ages of 3 and 10 [9,13,14,15,16,17].

The Numerical Rating Scale (NRS) is one of the most used pain scales. The NRS consists of a numeric type of the visual analog scale. The frequently used form of the NRS is a horizontal line with an eleven-point numbered range. It starts from zero to ten points, zero being associated with no pain and ten for the most intense pain possible. 

The Wong-Baker Faces Pain Rating Scale is the pain scale preferred by physicians and children. It is inexpensive and easy to use, even if measuring the pain for pediatric patients can be challenging. It consists of six faces that range from no pain to the worst pain. The faces range from smiling to grimacing. The patient matches the level of pain to a face on the scale. When this scale is used, children are able to communicate their level of pain, as opposed to surveys or other pain scales that give inaccurate results. The Wong-Baker Faces Pain Rating Scale seems to work best. 

The Functional Independence Measure (FIM) is a multidimensional questionnaire measuring the disability by evaluating motor and cognitive function (communication and cognitive/psychosocial). The motor function assessment of the FIM instrument refers to mobility and different activities of daily living skills, including eating, grooming, bathing, dressing the lower and upper body, toileting, toilet transfers, and in-home transfers. Total FIM scoring reflects all levels of dependence and an increased time needed for activities of daily living skills, or the use of assistive devices [13].

The follow-up was performed for a period of five years for all of the 15 pediatric patients included in the present study. Selection bias rarely happens in cohort studies, while the outcomes have not yet happened when the population is enrolled. A potential participant’s eventual outcome is unknown and cannot influence. Selection bias can appear in a prospective cohort study due to inequality in retention during the follow-up period after inclusion in the study. When the follow-up period spans many years (in retrospective or prospective cohort studies) it can be challenging to observe subjects for the entire study period. Many subjects fade due to death, relocation, or (in prospective studies) loss of concern in the study. Studies with follow-up rates lower than 60% will, in general, be perceived with limited validity. Still, even rates of 20% introduce bias if the causes for loss are correlated to exposure status and outcome status, respectively. Failure to follow-up can produce bias (an alteration of the observed value of the measure of relation from the value that would have been noted in the lack of a bias) if there are contrasts in the possibility of failure to follow-up associated with exposure status and outcome, respectively. Primary extensive prospective cohort studies are considered reliable if they can maintain follow-up of 80–90% of their sample for long periods.

Commonly, some of the individuals offered to be subjects in a prospective cohort study decline to attend. This generates a bias in retrospective cohort studies and case-control studies, while exposure status and outcomes have occurred at admission. Non-participations will not prejudice a prospective cohort study in which the event of interest has not yet taken place. For example, assume 100,000 subjects are invited to participate in a study regarding the correlation between smoking and heart attack where 50,000 decline to participate. Only 20% of those who participate were smokers, in contrast to 30% in the group who refused. The frequency of tobacco consumption in the study subjects does not represent the prevalence in the population. However, it can still analyze smokers and non-smokers without considering the incidence of myocardial infarction. In a behavior like this, significant non-participation could detract from the generalization of the observations. 

Data were statistically analyzed using Microsoft Excel 2013 as data editing and processing software. The statistical tools F-Test and Student’s *t*-test, integrated into the statistical software package, were used. A *p*-value of <0.05 was considered statistically significant. In addition, the F-test was used to verify whether the variance of the study group has equal variants, in order to decide which possible *t*-test to apply.

While osteogenesis imperfecta is a rare condition, a population of 15 participants is considered to be quite reasonable. A confidence level of 95% and a margin of error of 5% were taken into account while performing the statistical analysis. Therefore, the computed ideal sample size using the above parameters is 15. Assuming that all 30 patients taken first into account would match the inclusion criteria, the ideal population size computed would be 28 for 60 patients. Furthermore, the ideal population size would be 52. For 90 patients, n would be equal to 73, and for 120 patients, s n would be 92, respectively. Lastly, for 15 patients who were followed up for five years, the statistical conclusions should be reliable.

## 3. Results

The modified Bleck score results show that 40% from our study group are classified in the first three more affected categories:

1. Non-walker older than two years of age
2. Therapy walker with the help of crutches or canes
3. Therapy walker without the help of crutches or canes.

**Figure 3:**
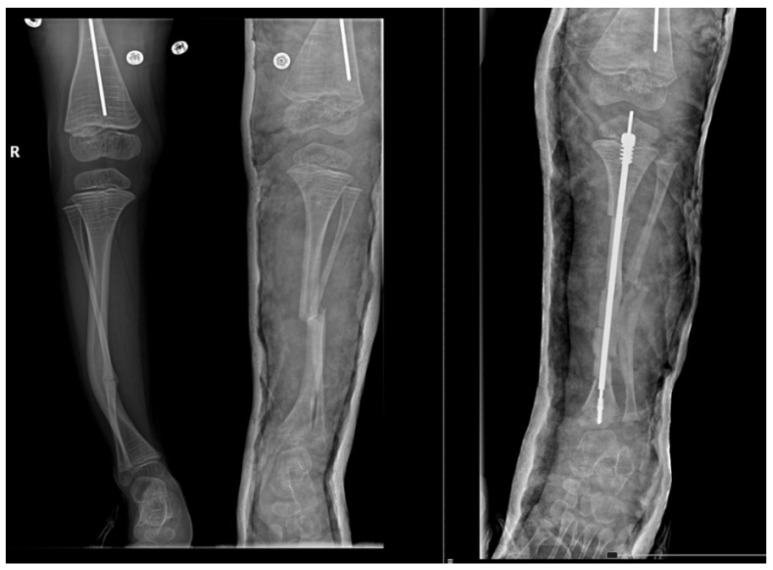
Before and after surgery aspects for a study participant.

Furthermore, we observed that the most considerable part of them, 33.33% had the first fracture diagnosed at childbirth (Osteogenesis imperfecta type III) and the other 6.67% noted the presence of fracture after birth (Osteogenesis imperfecta type I). Calculating Risk Ratio [RR = 3.33 (>1)] and Odds Ratio [OR = 6.25 (>1)], we can conclude that the type of Osteogenesis imperfecta is associated with the current capability of children to walk and their pattern of walking. No changes were perceived in the postoperative modified Bleck score compared to the preoperative results. As an example, a 5 year old pediatric patient diagnosed with Osteogenesis imperfecta Sillence type IV’s leg X-ray aspects from before and after the surgery is represented in Figure 3. 

For pain intensity assessment, we calculated the prevalence for each pain level, as seen in Table 1. 

Chronic pain was related to the whole body in addition to the surgical site. 

All patients present chronic pain fluctuating from mild to severe. 

After surgery, according to gender, 26.67% of answers recorded in the “mild pain” category were represented by females. 

For male patients, the most common response was “severe pain” (20%). 

The age group between 14–17 years has shown the highest percentage of “severe pain” compared to the other age groups. 

We calculated the average score of the FIM questionnaire in order to specify our study group ability, as seen in Table 2. 

From a maximum of 100%, the patients were described as 65% able after surgery (range 46–100%). A significant disability was observed in the following categories when combined with a lower FIM score: males, the patients living in rural areas and the group with the first fracture diagnosed at childbirth, or as we presumed the patients that identify as Osteogenesis imperfecta type III, accordingly to Sillence. However, no statistical significance was found.

## 4. Discussion

The most impaired categories, revealed by the modified Bleck score, are:

− non-walker older than two years of age
− therapy walker with the help of crutches or canes, and
− therapy walker without the help of crutches or canes.

All patients present chronic pain fluctuating from mild to severe. The age group between 14–17 years has shown a significant percentage of “severe pain” compared to the other age groups. A lower FIM score was a more significant disability in the following categories: males and the patients living in rural areas.

A critical aspect not often described in the evaluation of the quality of life, is chronic pain. The presence of intense chronic pain interferes with daily living activities, for instance, the self-care tasks of these patients. The quality of life can be improved overall after surgery but still remains a controversial topic.

Chronic and severe pain is a major complaint of the children diagnosed with Osteogenesis imperfecta decreasing their quality of life. In addition, pain intensity is strongly correlated with these children’s ability to perform the activities of daily living (ADL) and have a healthy cognitive function. 

Widmann, R.F. et al. [14] performed a cross-sectional study of 42 individuals with an average age of 33 diagnosed with Osteogenesis imperfecta. They used different questionnaires to assess physical and mental health limitations, comparing the results to the U.S. adult norms. Lower physical function scores were revealed, without significant differences in mental health. Besides, the study group showed high levels of educational achievement and employment. The FIM described the cohort as 97% able, being very functional despite musculoskeletal limitations. In our study, the FIM score increased after the surgical intervention from an average of 46% to 65%, still far from the results of Widmann et al.

R. Engelbert et al. [15] noted that functional abilities, mainly the mobility category, were associated with the Osteogenesis imperfecta subtype. Patients with a severe disease presented a lower score on mobility. Compared to the present study, it can be seen that even after surgery almost 20% of our cohort still remained with intense chronic pain.

All the patients we evaluated have impaired ambulation. However, due to the difficulty in differentiating between type I and III of Osteogenesis imperfecta, a small number of patients, and for other statistical reasons, we included the patients with the first fracture diagnosed in childbirth in class III and the rest in type I, as identified by Sillence. Consistent with the Bleck score, patients with type III, as identified by Sillence, showed a lower chance of independent walking than type I.

A total of 20% of patients belong to the first category of the Bleck classification, not walking. In addition, we observed that patients who achieved decent ambulation levels (Bleck score 7–9) were predominantly females. Also, assessing pain intensity, most recorded answers for “mild pain” were from female patients. 

The FIM score revealed a severe dysfunction in both physical and mental health. These results have been mostly seen in rural living patients suffering from difficulties in problem-solving and social interaction. There was no correlation found between the severity of motor and cognitive function impairment.

Some limitations can be observed, such as the small number of patients included in the study. Studies of larger population groups for a more extended follow-up period should be done to assess the quality of life and disability for patients diagnosed with osteogenesis imperfecta and treated with a telescopic rod. Further studies can compare and evaluate if early treatment with bisphosphonates can reduce chronic pain and the need for surgery.

Additional evaluation of the quality of life elements is fundamental in improving treatment procedures and social assistance care, supporting the category of children affected by osteogenesis imperfecta and their families.

## 5. Conclusions

Evaluating functional outcomes for patients with Osteogenesis imperfecta and telescopic rods, we noticed a significant ambulation impairment.

Treatment with bisphosphonates and surgery with telescopic rods are currently the gold standard for realigning bone deformities and multiple fractures. The results show an improvement regarding the quality of life, but the postoperative ambulation still remains to be improved. 

Generalized chronic pain still represents a significant problem involved in the treatment of pediatric patients diagnosed with Osteogenesis imperfecta.

The evaluation will be discussed in the following works. However, this study highlights the specific areas of quality of life affected in these pediatric patients in order to implement further appropriate measures that are intended to improve them.

## Figures and Tables

**Table 1:** Prevalence of pain level between males and females influencing activities of daily living before and after surgery.

	Preoperative				Postoperative		
	Pain level	Prevalence	Females	Males	Prevalence	Females	Males
	No pain	0%			0%		
−3	Mild pain (nagging, annoying, interfering little with ADL ^1^)	0%			40%	26.67%	13.33%
−6	Moderate pain (interferes significantly with A.D.L.)	60%	33%	26.66%	20%	6.67%	13.33%
−10	Severe pain (disabling; unable to perform A.D.L.)	40%	30%	30%	40%	20%	20%

^1^ The ability to perform the activities of daily living (ADL).

**Table 2:** Study group abilities based on the FIM questionnaire concerning sex, origin, and Sillence classification.

	Preoperative	Postoperative	
Sex	FIM Score ^1^	FIM Score ^1^	*p* ^2^
Males	45% able	63% able	*p* = 0.65
Females	47% able	67% able	
Origin			
Rural area	40% able		*p* = 0.58
Urban area		43% able	
Sillence classification			
Type III	64% able		*p* = 0.71
Type I	67% able		

^1^ FIM score was calculated before and after surgery. ^2^ *p*-value was calculated using the *t*-test.

## Data Availability

All of the data are registered at “Grigore Alexandrescu” Children’s Emergency Hospital, Bucharest, Romania.
